# Experimental Investigation of the Apparent Thermal Conductivity of Microencapsulated Phase-Change-Material Slurry at the Phase-Transition Temperature

**DOI:** 10.3390/ma14154124

**Published:** 2021-07-24

**Authors:** Krzysztof Dutkowski, Marcin Kruzel

**Affiliations:** Faculty of Mechanical Engineering, Koszalin University of Technology, ul. Raclawicka 15-17, 75-620 Koszalin, Poland; krzysztof.dutkowski@tu.koszalin.pl

**Keywords:** PCM, microencapsulation, thermal conductivity, experimental investigation

## Abstract

The article presents the results of detailed studies of the thermal conductivity of the water slurry of microencapsulated PCM (mPCM) and slurry based on water–propylene glycol solutions. The starting product, MICRONAL^®^ 5428 X, which contains about 43% microencapsulated paraffin with a transformation temperature of 28 °C, was mixed with the base liquid to obtain slurries with mass fractions of mPCM of 4.3, 8.6, 12.9, 17.2, 21.5, 25.8, 30.1, 34.4, 38.7, and 43.0%. Detailed measurements were carried out in the temperature range of 10–40 °C. It was found that: (a) an increase in the temperature of the slurry caused an increase in its thermal conductivity, both when PCM was in the form of a solid and a liquid; (b) the thermal conductivity of the mPCM slurry when the PCM was in liquid form was greater than the thermal conductivity of the slurry when the PCM was liquid; (c) during the phase transformation, a significant increase in the thermal conductivity of the slurry was observed, and its peak occurred when the temperature of the slurry reached the temperature declared by the manufacturer at which the phase-transition peak occurs.

## 1. Introduction

Thermal-energy storage (TES); e.g., of waste-heat or solar-radiation energy, is one of the ways to reduce greenhouse gas emissions. Intensive efforts are underway to reduce greenhouse gas emissions by at least 40% by 2030—the European Union’s policy goals set out in [1]. There are different ways to store energy [2] in the area of TES. The most important forms of TES are sensible heat storage (the heat input causes an increase in the temperature of the medium), latent heat storage (the heat input causes a phase change of the medium practically at its constant temperature), and thermochemical heat storage (materials that allow a reversible, exothermic chemical reaction).

### 1.1. Phase-Change Material and Working Fluids in Heat-Exchange Systems

The study of the properties of phase-changing materials (PCMs) as potential latent heat stores was initiated by Telkes and Raymond in the 1940s during an energy crisis, when new ways of storing heat, especially energy, were sought, especially solar energy [3]. PCMs, in addition to the ability to store large amounts of latent heat (in relation to units of mass), should be characterized by a high thermal-conductivity coefficient. A high value guarantees a quick “charging/discharging” process of the heat storage [4,5]. Organic (paraffinic) and inorganic (fatty acids, molten salts, glycols) materials, as the most common PCMs, are characterized by a low thermal-conductivity coefficient. Actions are underway to increase the value of this coefficient. This can be achieved through [6]: (a) placing the PCM material in a porous structure with a high thermal-conductivity coefficient (e.g., metallic foams, expanded graphite); (b) direct addition to PCM of materials with a high thermal-conductivity coefficient (e.g., copper, iron nanoparticles); or (c) PCM encapsulation [7]. One of the advantages of encapsulation is the possibility of simultaneous initiation of the phase-change process of the enclosed material, thanks to the thermal energy transferred from the intermediate fluid washing the capsules. The smaller the size of the capsule, the more zones in which the process of heat exchange and phase change takes place. Technological progress has made it possible to use a thin layer of PCM particles with sizes on the order of micro- (mPCMs) and even nanometers (nPCMs) for coatings [8,9]. With these capsule sizes, it is possible to flow not only the rinsing liquid, but the entire mPCM/nPCM slurry. The resulting slurry, in addition to ice slurries or emulsions composed of PCMs directly added to the base liquid, belongs to the group of the so-called latent functional thermal fluids (LFTFs) [10,11]. LFTFs can be used not only as a heat store, but also as a heat carrier in heat-exchange systems. The main advantages of using mPCM/nPCM slurries in heat-exchange systems include the possibility of reducing the amount of working fluid, the diameter of the channels, the heat-exchange surface of the exchangers, or the amount of energy required to pump the fluid [12,13]. Numerous tests have been carried out to determine the flow-resistance or heat-transfer coefficients [14,15,16] during the flow of mPCM and nPCM slurries in pipe minichannels. The assessment of the possibility of using the relations used so far to calculate the dimensionless heat-transfer coefficient (Nusselt number) for mPCM/nPCM slurries requires precise knowledge of the value of the heat-conductivity coefficient, especially in the slurry’s operation range; i.e., the PCM’s phase-transition temperature range.

There are many techniques for measuring the heat-conduction coefficient of PCM materials and materials containing PCM additives (composites, encapsulated powders, mPCM slurries), which can be divided into the guarded hot plate, heat-flow-meter methods, and transient methods (e.g., DSC, hot ware, laser flash, etc.) [6]. However, there is no method that could be considered the most suitable for measuring the thermal conductivity of mPCM slurries. This is due to the lack of comparative measurements carried out with different methods using the same sample [6]. Moreover, none of the above-mentioned methods has been applied to the measurement of thermal conductivity at the temperature at which PCMs undergo a phase change. Therefore, it is not possible to obtain the results of experimental studies of thermal conductivity during the phase transition of PCMs, and hence attempts to create appropriate mathematical relationships.

### 1.2. Thermal Conductivity of Micro-/Nanoencapsulated PCM Slurries—Previous Research

In a paper by Zhang and Zhao [17], the authors presented the results of experimental studies of thermal and rheological properties of a slurry composed of a base liquid (water), microencapsulated PCM, and a surfactant. The products used as PCMs were those with the trade designations DPNT06-0182 (Ciba Specialty Chemicals) and Micronal DS 5008X (BASF). The tests were carried out only at one slurry temperature (20 °C) and three mass fractions of the PCM: 10, 25, and 35%, respectively. It was observed that with increasing concentration of microcapsules, the thermal conductivity of the slurry decreased because the thermal conductivity of PCM was lower than that of the base liquid. A similar conclusion followed from the subsequent experimental work carried out at one temperature of the slurry [18,19], or two different: one measurement was made when the PCM was a liquid, and one when it was a solid [20]. The authors of [20] found that when the PCM (n-octadecane) was solid, the value of the thermal conductivity of the slurry was lower than when the PCM was a liquid.

Delgado et al. [21] dealt with microencapsulated aqueous slurry of a PCM (polymer-coated paraffin). The mass fractions of the PCM were 14, 20, and 30%. The PCM was characterized by a phase-transition temperature of about 20–24 °C. Measurements of the heat-conductivity coefficient were carried out for three temperature values: 20, 25, and 30 °C. The higher the proportion of PCM in the slurry, the lower the values of the thermal-conductivity coefficient, regardless of the sample temperature. It was difficult to determine the influence of the sample temperature on the thermal-conductivity coefficient, as it increased for a concentration of 14% (and slightly for 20%), and decreased for a concentration of 30%.

Five different temperature values (25, 28, 30, 32, and 34 °C) of the aqueous slurry of nanocapsulated PCM (n-nanodecane) were used to determine the thermal conductivity coefficient in the work by Karaipekli et al. [11]. The phase-transition temperature of n-nanodecane was 32 °C. As the temperature of the sample increased, the value of the thermal-conductivity coefficient increased, and the growth rate increased as the temperature of the slurry rose. Contrary to paraffin, an increase in the mass fraction of the PCM (from 0.5% to 2.0%) resulted in an increase in the thermal-conductivity coefficient, regardless of the sample temperature.

Measurements of the thermal conductivity of the slurry at six different temperature values of the sample were described in several subsequent works. Wang L. et al. [22] conducted tests at temperatures of 20, 30, 40, 50, 60, and 70 °C using a microencapsulated PCM slurry with a phase-change temperature of approximately 54 °C. An increase in the mass fraction of mPCM (from 10% to 40%) in the base liquid (a mixture of water and propanol) resulted in a decrease in the thermal-conductivity coefficient. An increase in sample temperature caused an increase in the thermal-conductivity coefficient, the maximum value of which (peak) was reached at a temperature of 40 °C. A further increase in the sample temperature caused a decrease in the thermal-conductivity coefficient. Wang F. et al. [23] carried out tests at 30, 40, 50, 60, 70, and 80 °C for emulsions of PCM (paraffin) with a phase-transition temperature of about 63 °C. The increase in the mass fraction of the PCM (from 10% to 30%) in the base liquid (a mixture of polyvinyl alcohol and polyethylene glycol) resulted in a decrease in the thermal-conductivity coefficient. The thermal conductivity of the emulsion increased slowly with increasing temperature and then increased sharply, reaching a maximum near the melting point of the PCM. A temperature increase above the phase-transition temperature (~60 °C) to 70 °C caused a sharp decrease in thermal-conductivity value. At this temperature, complete melting of the PCM material was achieved, and further heating of the sample (up to 80 °C) caused a slight, almost imperceptible increase in the thermal-conductivity coefficient. It should be noted that the thermal conductivity of the emulsion when the PCM was a liquid was lower than when the PCM was solid. Similar behavior of the slurry was described in [10], which reported the results of research on microencapsulated paraffin/melamine resin as an addition to a mixture of water and ethanol, and the work presented in [24], in which the results of testing the properties of aqueous microencapsulated slurry of n-eicosane were described. Xu et al. [25] investigated the thermal conductivity of an aqueous slurry of microencapsulated paraffin containing the addition of Cu, Cu_2_O nanoparticles, and carbon nanotubes (CNT). The paraffin’s phase-transition temperature was 62 °C, and the tests were carried out in a temperature range of 30–80 °C. The thermal conductivity of the slurry proportionally increased with increasing temperature. There was no apparent effect of the phase change on the thermal conductivity of the slurry.

The study of natural convection from the horizontal heating surface to the mPCM slurry was described in [26]. The proper tests were preceded by determining the properties of the working fluids—several kinds of n-paraffin waxes in water. The phase-transition temperature of the paraffin was 31 °C, and the tests of the heat-conductivity coefficient were carried out in a temperature range of 5–50 °C. It was noted that an increase in the sample temperature to about 20 °C resulted in a slight increase in the thermal-conductivity coefficient. The higher the proportion of mPCM in the slurry, the lower the values of the thermal conductivity. Further heating of the sample to a temperature of about 38 °C caused a decrease in the thermal-conductivity coefficient. The thermal conductivity obtained at this temperature (the end of the PCM phase transformation) was lower than when the PCM was solid. The higher the share of PCM in the slurry, the higher the rate of decrease in the value of the thermal-conductivity coefficient. Further heating of the sample resulted in a slight increase in the thermal-conductivity coefficient again.

Detailed studies of the thermal conductivity were described by Allouche et al. [27]. The thermal conductivity of an aqueous slurry of wet encapsulated paraffin with a phase-change temperature of 15 °C was determined in a temperature range of 5 °C to 25 °C. Dozens of measurements were made while both increasing and decreasing the sample temperature. Increasing the temperature of the slurry (when the PCM was a solid) increased the thermal conductivity of the slurry, and the growth rate rose as the temperature approached the phase-transition temperature. After exceeding this temperature, when the phase-transition process was completed, the value of the thermal-conductivity coefficient dropped abruptly and fell to a value lower than when the PCM was a solid. Further heating of the sample did not change the value of the thermal conductivity of the slurry. A slight hysteresis of the heat-conduction coefficient was noticed at the phase-change temperature during the heating and cooling of the sample. An analogous course of changes in the thermal-conductivity coefficient was observed during the tests of an emulsion composed of 16% paraffin added to water [28]. Detailed measurements of the thermal-conductivity coefficient in the temperature range of 15–30 °C showed that it reached the highest value when the paraffin’s phase-transition temperature (23 °C) was reached. The value of the thermal conductivity of the emulsion when the PCM was solid was close to that of an emulsion when the PCM was a liquid. The increase in sample temperature resulted in a slight increase in the thermal conductivity of the emulsion in both PCM states.

The following conclusions can be drawn from the literature review: (a) the thermal conductivity coefficient of slurry/emulsions containing PCM is determined while conducting specific tests (e.g., of heat transfer), hence it is determined only for a few selected temperature values; (b) the most frequently described tests of the properties of mPCM slurries concern the determination of the influence of the mass fraction of the PCM in the slurry on its thermal or rheological properties; (c) there are few publications in which the problem of thermal conductivity of slurries has been studied in more detail, especially in terms of the phase-transition temperature of the PCM; (d) the few detailed studies do not systematize how the material of the base liquid, especially PCM-capsule vapor, affects the thermal conductivity of the slurry when the PCM is a liquid, solid, or undergoing a phase change.

This article describes the results of detailed testing of a slurry consisting of the product MICRONAL^®^ 5428 X added to water and an aqueous solution of propylene glycol. The mass fraction of the product in the slurry varied from 10 to 100% (every 10%). As the product itself contains about 43% microencapsulated paraffin, the proportion of PCM in the tested slurries was respectively: 4.3, 8.6, 12.9, 17.2, 21.5, 25.8, 30.1, 34.4, 38.7, and 43.0%. The temperature of the phase transformation (melting) of the paraffin was 28 °C, and the measurements of the heat-conductivity coefficient were carried out in a temperature range of 10–40 °C.

## 2. Methodology and Materials

### 2.1. Preparation of the Slurry

A total of 18 samples of the slurry were prepared. In nine of them, the base liquid was distilled and demineralized water. In the next nine samples, an aqueous propylene glycol solution with the trade name ERGOLID EKO^®^ was used as the base liquid. Propylene glycol solution is a fluid used, inter alia, in liquid installations of solar collectors, and prevents the liquid from freezing in winter conditions. The solution contained 37% propylene glycol, a set of inhibitors protecting against corrosion of the solar collector system material, and biocides preventing the formation of biological life. The rest of the solution was water.

An additive to the base liquids was a product of Microtek Labs (Moraine, OH, USA) under the trade name MICRONAL^®^ 5428 X. The main components of the product, according to the manufacturer’s data [29], were paraffin wax microencapsulated in a highly crosslinked polymethylmethacrylate polymer wall and water. The mass fraction of mPCM in water was 43 ± 1 wt %. The mean peak of the melting point of paraffin was 28 °C ± 1 °C, and the heat of fusion was ≥160 kJ kg^−1^.

The test samples were made by adding the original slurry to the base liquid, and then the whole was stirred mechanically for 15 min. A homogeneous slurry was obtained. As the share of mPCM in the original product was on average 43%, and the product was added to the base liquid in the mass fractions of 10, 20, 30, 40, 50, 60, 70, 80, and 90%, when the slurry samples were obtained, the mass fraction of mPCM was respectively: 4.3, 8.6, 12.9, 17.2, 21.5, 25.8, 30.1, 34.4, 38.7, and 43.0% (pure MICRONAL^®^ 5428 X). The mass fractions of the main components of the slurry when MICRONAL^®^ 5428 X was added to the aqueous propylene glycol solution are summarized in Table 1.

### 2.2. Experimental Facility

The thermal conductivity of the test slurry was measured with a TEMPOS Thermal Analyzer from METER Group, Inc., Pullman, WA, USA (successor of the KD2 PRO commonly used in thermal research). The meter was equipped with a KS-3 measuring probe, a single-needle probe 60 mm long and 1.3 mm in diameter. The determination of the thermal conductivity coefficient was based on the transient state method. The measurement consisted of several steps. Initially, a low-power heat source in the probe needle generated a heat pulse. The low power of the heat source prevented convection of the free fluid sample. Then a temperature sensor, located in the same needle, recorded the temperature response of the system. The temperature changes recorded by the instrument were the basis for determining the thermal conductivity of the material surrounding the probe needle. The whole cycle lasted 60 s, and the accuracy of a single measurement declared by the manufacturer is ±10%.

**Figure 1:**
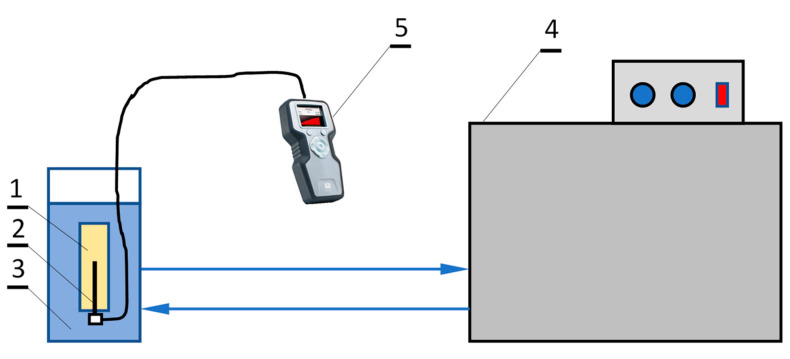
Diagram of the test stand; 1—tested slurry, 2—probe needle, 3—water bath, 4—cryostat, 5—TEMPOS thermal properties analyzer (METER Group, Inc., Pullman, WA, USA).

The tested mPCM slurry was contained in a vial with the dimensions of a standard vial with glycerin—verification liquid attached to the kit (d = 28 mm, L = 100 mm). The needle of the measurement probe was inserted through the stopper in the center of the vial. The whole was placed vertically, upside down, in a vessel with a water bath (Figure 1). The needle was completely immersed in the test liquid. Water was circulating between the cryostat and the vessel in which the sample and measuring probe was placed. The cryostat made it possible to smoothly adjust the temperature of the circulating water, and thus the tested slurry.

### 2.3. Procedure of the Experimental Research and the Conditions of the Experiment

A vial with a slurry of known mPCM concentration was placed in a water bath. The sample was then cooled down to about 10 °C. After reaching a steady state, the actual experiment was started. The water temperature was gradually increased. When a certain value was reached, heating was discontinued, and the sample was allowed to stop changing temperature and then measured with a thermal analyzer. The analyzer measured the current sample temperature and the value of the thermal-conductivity coefficient. Measurement of the thermal conductivity of the slurry was carried out for several dozen different temperatures in the range of 10–40 °C. In this way, for a given concentration of the sample, a series of measurements was obtained showing the change in the thermal-conductivity coefficient. The series of measurements was repeated twice more, and the presented results of thermal conductivity constituted the arithmetic mean of the values obtained for a given temperature of the mPCM slurry. The average temperature value was determined with an accuracy of ±0.2 °C.

## 3. Results and Discussion

**Figure 2:**
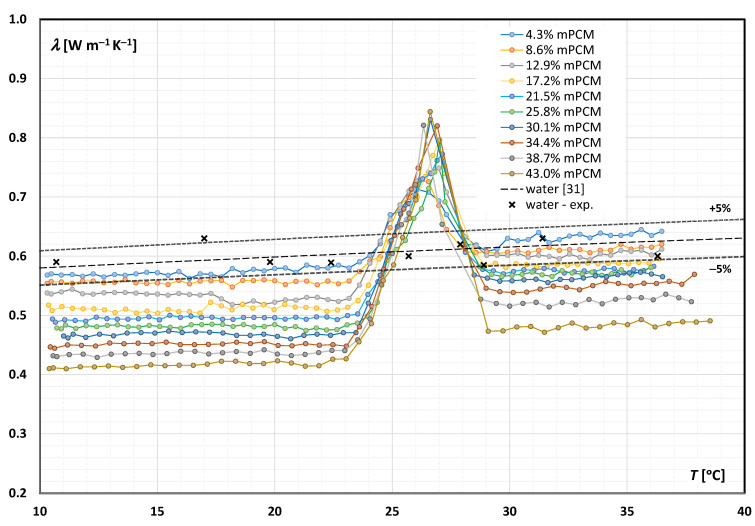
Thermal conductivity of mPCM aqueous slurry as a function of temperature.

Figure 2 presents a summary of the results of the research on the thermal-conductivity coefficient (*λ*) of an aqueous mPCM slurry in a range of temperatures. Each curve represents a slurry with a different mass fraction of mPCM. In addition to the experimental data obtained for the tested slurry, it includes experimental data of the conductivity coefficient obtained during several test measurements with distilled water, and curves—data for water according to Standard Reference Data from the NIST [30] (Table 2), and curves representing values 5% greater/less than the reference values. It was noted that the own experimental data obtained for water were comparable with the data from the literature. Hence, it was concluded that the applied method and test procedure were appropriate for the correct determination of the slurries’ thermal conductivity.

It can be seen from Figure 2 that an increase in the mass fraction of mPCM in the slurry caused a decrease in the thermal conductivity of the slurry. This is because the microencapsulated paraffin had a thermal conductivity much lower than the thermal conductivity of water. Hence, the lower the mPCM content in the slurry, the higher the thermal conductivity of the slurry, and it tended toward the value of the thermal conductivity of the base liquid—water. The trend obtained for the concentration of 12.9% was an exception, for which one of the three measurement series gave slightly different results, but dominated the mean on the basis of which the characteristics were prepared.

It was noted that when the proportion of mPCM in the slurry was 4.3% and T > 29 °C, the measured thermal conductivity of the slurry was greater than for the reference liquid—water. This may be due to the fact that other chemicals (e.g., surfactants) were present in the dispersible product, which may have caused all of the results obtained to be slightly higher than those obtained with a slurry composed of microencapsulated paraffin and pure water.

It was noted (Figure 2) that an increase in the temperature of the slurry caused an increase in the value of the thermal conductivity coefficient, both when the PCM was in the form of a solid (T = 10–23 °C) and when the PCM was a liquid (T = 29–40 °C). For these temperature ranges, the course of the characteristics of the thermal-conductivity coefficient from temperature was comparable to the course of the characteristics of the base liquid (water), which was the dominant component of each of the tested slurry samples. The values of the thermal conductivity of the slurry when the PCM was a liquid were higher than the values of thermal conductivity when the PCM was a solid, regardless of the mass fraction of the mPCM. Since microencapsulated solid paraffin has a higher thermal conductivity than microencapsulated liquid paraffin [31], the trend obtained was surprising. This was explained by the fact that the share of paraffin mass in the slurry was smaller than that of the base liquid, hence the thermal conductivity of the base liquid was dominant. The analysis of the literature showed that a similar tendency was also noticed in the works [23,24,28].

A clear increase in the value of the thermal-conductivity coefficient occurred when the PCM underwent a phase change. It was an apparent thermal conductivity because the heat pulse generated during the test in the measuring probe caused only a slight increase in the internal energy of the slurry sample, which was also the case in materials with high heat conductivity in the volume. This was because some of the heat input caused a phase change for the PCM particles closest to the probe. It should be noted that the peak thermal conductivity was reached when the highest number of PCM particles had undergone a phase change. The maximum value of the thermal-conductivity coefficient was approximately λ ≈ 0.84 W m^−1^ K^−1^, and it was reached at a phase-transition temperature T = 27 °C. The higher the concentration of mPCM in the slurry, the greater the peak that was observed—the increase in the value of thermal conductivity during the phase transition of PCM. Characteristically, the thermal conductivity increased gradually (not much) as the first PCM particles began to undergo a phase change (around 23 °C), and dropped sharply once the PCM was liquid. A slow increase and a sharp decrease in thermal conductivity in the range of the PCM phase-transition temperature was also observed in the works [23,27,28,32].

**Figure 3:**
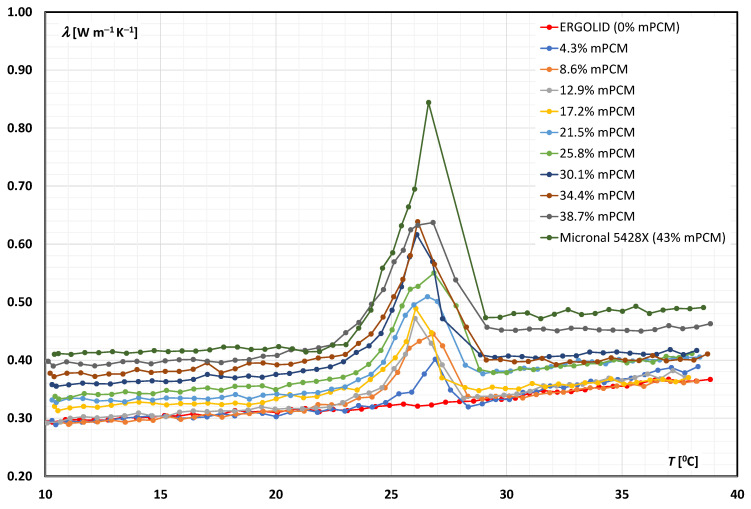
Thermal conductivity of mPCM slurries based on aqueous propylene glycol as a function of temperature.

Figure 3 presents the results of tests of the thermal-conductivity coefficient *λ* of the mPCM slurries (based on an aqueous solution of propylene glycol) as a function of temperature.

Each curve represents a slurry with a different mass fraction of mPCM, propylene glycol, and water (Table 1). It was noted that the analogy between the results presented for the aqueous mPCM slurry (Figure 2) was maintained: (a) an increase in the temperature of the slurry caused an increase in its thermal conductivity; (b) the thermal conductivity of the slurry was greater when the PCM was a liquid; (c) in the phase-transition temperature range, the apparent conductivity of the slurry slowly increased and then dropped sharply after exceeding 27 °C; and (d) the peak of increase in thermal conductivity was greater as the mPCM content in the slurry rose, but there was one fundamental difference. Contrary to the case of an aqueous mPCM slurry, an increase in the mass fraction of the mPCM caused an increase in the thermal conductivity of the slurry. This was due to the fact that the aqueous propylene glycol solution had a lower thermal conductivity (λ ≈ 0.3–0.36 W m^−1^ K^−1^—Figure 3.) than the added aqueous mPCM slurry (λ ≈ 0.4–0.5 W m^−1^ K^−1^—Figure 3.). Therefore, the more water there was in the slurry, the greater the conductivity of the slurry.

### 3.1. Experimental vs. Theoretical Thermal Conductivity of the mPCM Slurry

The theoretical thermal conductivity of a slurry with the addition of nano- or microencapsulated PCM is usually determined using the Maxwell equation of the form [22,33,34,35,36]:

(1) $$\frac{\lambda_{sl}}{\lambda_{bf}}=\frac{\lambda_{bf}+2\lambda_{k}+2\phi \left(\lambda_{k}-\lambda_{bf}\right)}{\lambda_{bf}+2\lambda_{k}-\phi \left(\lambda_{k}-\lambda_{bf}\right)}$$

where *ϕ* denotes the volume fraction of the dispersed phase (capsules) in the base liquid, and the indices *sl*, *bf*, *k* denote the slurry, base liquid, and capsules, respectively. The model can be successfully used when the capsules have a spherical shape, and its usefulness is less when the shape of the dispersed phase particles differs from the spherical shape [37,38]. It should be emphasized that the experimental studies showed that the thermal conductivity of the mPCM slurry when at rest was lower than when the slurry was in the flow. In order to calculate the theoretical heat-conduction coefficient mPCM in the flow, in addition to the calculations from the Maxwell formula, additional calculations should be made while taking into account the movement of PCM particles [39,40,41].

There is a component in Equation (1), $\lambda_{k}$—the heat-conduction coefficient of the PCM microcapsule. It is calculated assuming that the dispersed phase consists of multilayer spheres. The thermal conductivity of such a ball can be calculated from the thermal conductivity of the core material (*λ_c_*), shell (*λ_s_*), particle diameter (*d_k_*), and the diameter of the particle core (*d_c_*) of the mPCM according to dependencies [34,36,39,40,41]:

(2) $$\frac{1}{\lambda_{k}d_{k}}=\frac{1}{\lambda_{c}d_{c}}+\frac{d_{k}-d_{c}}{\lambda_{s}d_{k}d_{c}}$$

As the diameter of the multilayer ball changes with temperature, the ratio of the diameter of the ball and its core depends on the density of the shell material (*ρ_s_*) and the density of the core material (*ρ_c_*) in the relationship [38,40]:

(3) $${\left(\frac{d_{c}}{d_{k}}\right)}^{3}=\frac{\rho_{s}}{\rho_{s}+x\rho_{c}}$$

where *x* is the core–shell weight ratio.

**Figure 4:**
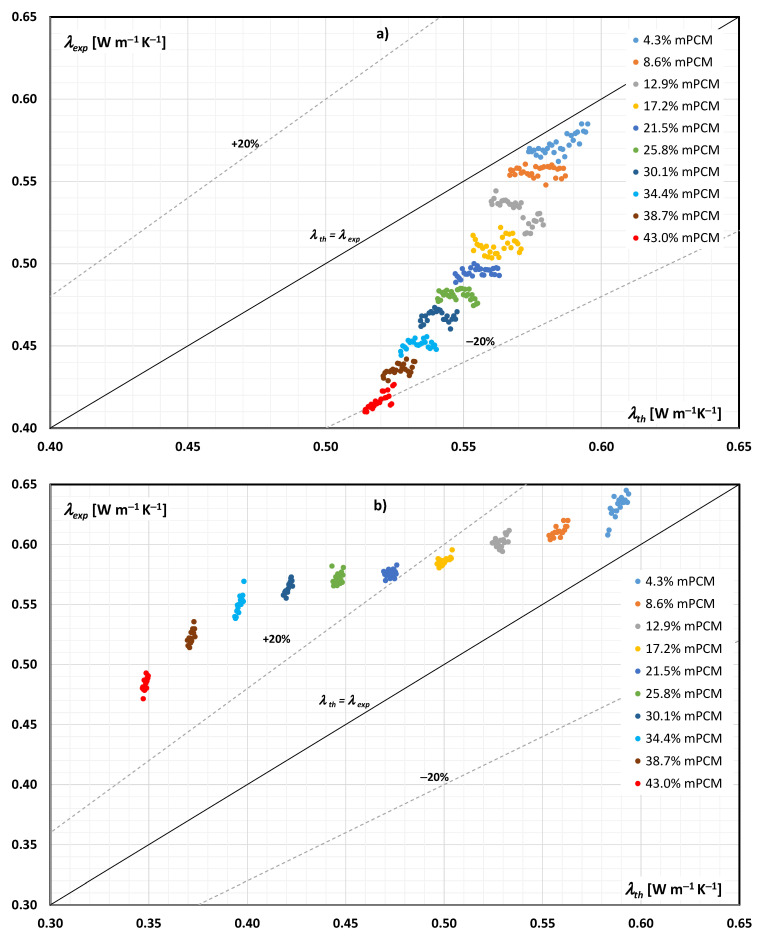
Comparison of the experimental and theoretical (according to Maxwell’s dependence) thermal conductivity of the aqueous mPCM slurry: (**a**) PCM in the form of a solid, (**b**) PCM in the form of a liquid.

**Figure 5:**
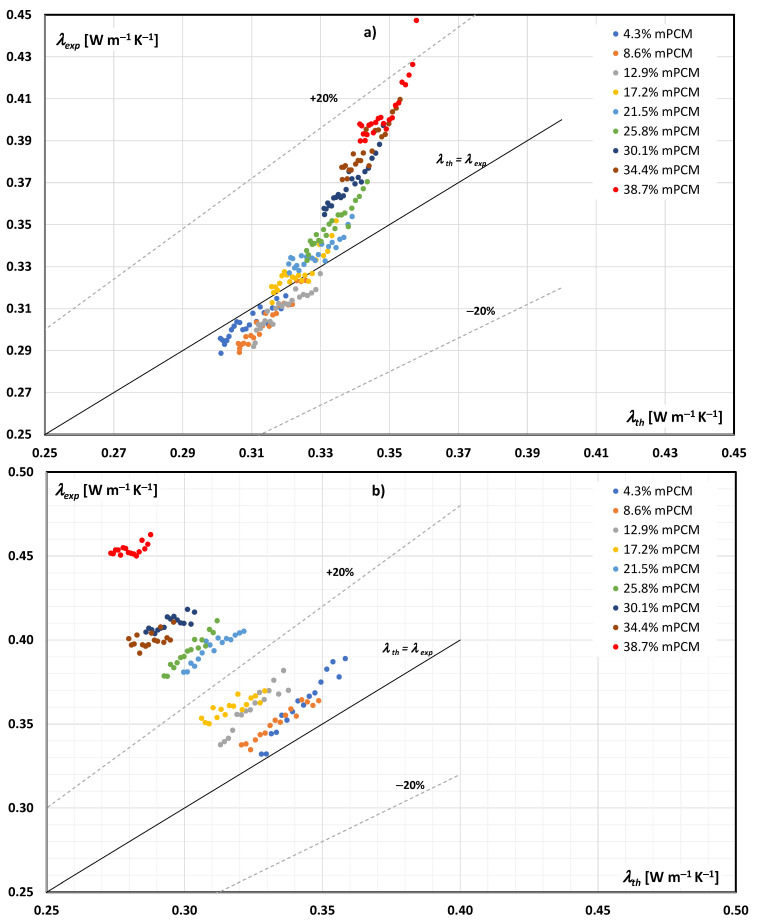
Comparison of the experimental and theoretical (according to Maxwell’s dependence) thermal conductivity of mPCM slurry based on an aqueous solution of propylene glycol: (**a**) PCM in the form of a solid, (**b**) PCM in the form of a liquid.

Figure 4 and Figure 5 present a comparison of the results of experimental tests of the thermal conductivity of the mPCM slurry with the results of calculations according to Equations (1)–(3). The data in Figure 4 are for an aqueous mPCM slurry, while the data in Figure 5 are for an mPCM slurry based on an aqueous propylene glycol solution. It should be emphasized that the data presented in Figure 4a and Figure 5a concern the case when the PCM in microcapsules was in the form of a solid (slurry temperature below 23 °C), while the data in Figure 4b and Figure 5b apply to the case when the PCM in microcapsules was in the form of a liquid (slurry temperature above 29 °C).

Figure 4 and Figure 5 additionally contain auxiliary lines. The solid line reflects the case when *λ_exp_* = *λ_th_*, and the dashed lines indicate the cases when *λ_exp_* is greater/smaller than the theoretical value by 20%.

An analysis of Figure 4a shows that when the PCM in the mPCM slurry was in the form of a solid, its thermal conductivity could be determined based on the Maxwell equation with an error not greater than 20% in the entire studied range. When the PCM was in a liquid form (Figure 4b), Maxwell’s equation underestimated the thermal conductivity of the slurry when the mPCM concentration exceeded 21.5%.

An analysis of Figure 5a shows that when the PCM in the mPCM slurry was in the form of a solid, its thermal conductivity could be determined based on the Maxwell equation with an error not greater than 20% in the entire studied range. What changed if the PCM in the microcapsules was in the form of a liquid? Already at 21.5% mPCM concentration in the base liquid, the experimental thermal conductivity coefficient reached values much higher (over 20%) than the results from the Maxwell equation. The research results indicated that if the PCM in the slurry was in the form of a liquid, the applicability of the Maxwell equation was limited to slurry in which the mPCM content did not exceed about 20%.

Table 3 provides a summary of the mean absolute percentage error (MAPE), calculated by [42,43,44,45]:

(4) $$\mathrm{MAPE}\%=\frac{100}{N}\cdot \overset{N}{\underset{1}{\sum }}\left(\frac{\left|\lambda_{th}-\lambda_{exp}\right|}{\lambda_{exp}}\right)$$

where *N* is the number of measurements. From the analysis of the obtained results, it was concluded that when the PCM was in the form of a solid, the MAPE error varied from 2.1% to 24.7% for the mPCM content in the aqueous slurry from 4.3% to 43%, and from 2.1% to 13.7% when the mPCM content in the slurry of the aqueous glycol solution varied from 4.3% to 38.7%. Similarly, when the PCM was in the liquid form, the MAPE error changed from 6.8% to 28.0% for the mPCM content in the aqueous slurry from 4.3% to 43%, and from 5.1% to 37.3% when the mPCM content in the aqueous glycol slurry changed from 4.3% to 38.7%.

The conducted research and theoretical analyses showed that when the PCM (paraffin) filling the microcapsules was in the form of a solid, it was possible to use the Maxwell equation to calculate the thermal conductivity of both the water-based slurry and the water-based propylene glycol slurry. The error resulting from the application of Equations (1)–(3) decreased with the decrease in the mass fraction of the mPCM in the slurry. The application of the Maxwell equation to mPCM slurry in which the paraffin is in the liquid form is limited. For the tested slurries, the theoretical error in determining the thermal conductivity coefficient will be less than 20%, when the mass fraction of mPCM in the slurry does not exceed 20%. Observations regarding the limited or inability to use the Maxwell equation to calculate the thermal conductivity of a slurry when PCM is in liquid form, based on numerous publications, are included in the review article [35].

### 3.2. Discussion of Research Results

The literature review found no results of basic tests of properties (including the thermal-conductivity coefficient, *λ*) of mPCM slurry at the phase-transition temperature or the influence of other values on the value of these properties. The presented research results fill the information gap and relate to the previously unrecognized influence: temperature (in the scope including the temperature immediately before, during, and after the PCM phase change), the aggregate state of the phase-change material enclosed in the capsule (solid/two-phase mixture/liquid), the type of base liquid (water and an aqueous solution of propylene glycol), and the mass fraction of microcapsules (up to 43%) in the base liquid on the thermal-conductivity coefficient of the slurry. The test results were obtained for nine different concentrations of mPCM slurry.

It was noted that for slurries based on a commonly used liquid (water), an increase in the temperature of the slurry caused an increase in the value of the thermal-conductivity coefficient, and the growth rate (*λ*) was the same as that with water as the base liquid. Thus, the base liquid had a dominant influence on the value of the thermal-conductivity coefficient (*λ*) of the mPCM slurry. This was not the case with the temperature range in which the PCM material absorbed the heat necessary to change the physical state. Then a clear peak was observed—a sharp increase and then a sharp decrease in the value of the thermal-conductivity coefficient. As a result, the thermal-conductivity coefficient reached a level similar to that before the phase transition began. The increase in the value of the heat-conductivity coefficient (peak) depended on the mass fraction of the mPCM, and reached about 100% for the slurry with the highest mass fraction of mPCM (43%). Thus, in terms of the phase transformation, the PCM material encapsulated in the microcapsules had a decisive influence on the value of the phase-transformation ratio. The influence of the mass fraction of mPCM on the value of the thermal-conductivity coefficient when the PCM was in a single-phase state also was noticed. Then, regardless of the temperature of the slurry, an increase in the proportion of mPCM caused a decrease in the value of the thermal conductivity of the slurry. The situation was similar for mPCM slurries based on an aqueous solution of propylene glycol. An increase in the temperature of the slurry caused an increase in the value of the heat-conductivity coefficient, and the rate of changes was analogous to that of the base liquid. Similar to the water-based slurry and the slurry based on an aqueous propylene glycol solution, a clear peak of the value of *λ* was observed in the phase-transition-temperature range. The instantaneous increase in the value of *λ* also reached 100%. Contrary to the water-based slurry, this time, the greater the proportion of microcapsule mass in the aqueous propylene glycol solution, the greater the thermal conductivity of the slurry. Therefore, it was concluded that the type of the base liquid, the mass fraction of mPCM, and the temperature significantly impacted the value of the thermal-conductivity coefficient. Unknown in detail from the literature, we showed the phenomenon of a rapid increase and then a decrease (peak) in the value of the heat-conductivity coefficient of the slurry when the PCM underwent a phase change, and the influence of the mPCM on its value. It has been shown that an increase in mPCM in the slurry may result in a decrease or, on the contrary, an increase in the value of *λ*, depending on the nature of the base liquid.

Based on the comparison of numerous results of experimental tests and calculations, it has been shown that it is not possible to use Maxwell’s relationship to determine the value of the thermal conductivity coefficient when PCM undergoes a phase change. To a limited extent, this equation is applicable when the PCM in the microcapsules is in the form of a liquid. It is allowed to use the Maxwell equation to determine the thermal-conductivity coefficient of mPCM slurries when the phase-change material is in the liquid state. However, it should be borne in mind that the higher the concentration of mPCM in such a slurry, the greater the error of the calculation results. In terms of the research carried out here, this error reached 20%.

## 4. Summary and Conclusions

Based on experimental tests carried out in the temperature range of 10–40 °C with the use of an aqueous slurry of mPCM (paraffin in the PMMA shell) and a slurry based on an aqueous solution of propylene glycol with mass fractions of mPCM of 4.3, 8.6, 12.9, 17.2, 21.5, 25.8, 30.1, 34.4, 38.7, and 43.0%, the following conclusions were drawn:

- An increase in the temperature of the slurry caused an increase in its thermal conductivity, both when the PCM was in the form of a solid and a liquid;
- The thermal conductivity of the mPCM slurry when the PCM was in liquid form was greater than the thermal conductivity of the slurry when the PCM was solid;
- During the phase transformation, a significant increase in the thermal conductivity of the slurry was observed, and its peak took place when the temperature of the slurry reached the temperature declared by the manufacturer at which the phase-transition peak occurs;
- The thermal conductivity value (apparent thermal conductivity) obtained during the phase transformation did not correspond to the actual value, but results from the imperfection of the measurement method;
- The thermal conductivity of an mPCM slurry can be determined from Maxwell’s dependence with an accuracy of ±20% when the PCM is in the form of a solid in the entire tested range, and to a limited extent (mPCM mass share up to 20%) when the PCM is in liquid form in the slurry;
- Work is required to set standards for measurement methods or standardization of the preparation of mPCM slurry samples in order to measure their thermal conductivity, especially at the temperature at which the PCM undergoes a phase change.

## Figures and Tables

**Table 1:** Mass fractions of major components of mPCM slurry based on aqueous propylene glycol solutions.

Mass Fractions of Slurry Components (%)		
mPCM	Glycol	Water
43.0	0	57.0
38.7	3.7	57.6
34.4	7.4	58.2
30.1	11.1	58.8
25.8	14.8	59.4
21.5	18.5	60.0
17.2	22.2	60.6
12.9	25.9	61.2
8.6	29.6	61.8
4.3	33.3	62.4
0	37.0	63.0

**Table 2:** Thermal-conductivity coefficient data for water according to Standard Reference Data—NIST [30].

Temperature (°C)	Thermal Conductivity (W m^−1^K^−1^)	Temperature (°C)	Thermal Conductivity (W m^−1^K^−1^)	Temperature (°C)	Thermal Conductivity (W m^−1^K^−1^)
10.0	0.58005	20.0	0.59846	30.0	0.61550
10.5	0.58099	20.5	0.59935	30.5	0.61631
11.0	0.58193	21.0	0.60024	31.0	0.61711
11.5	0.58287	21.5	0.60112	31.5	0.61790
12.0	0.58380	22.0	0.6020	32.0	0.61869
12.5	0.58474	22.5	0.60288	32.5	0.61948
13.0	0.58567	23.0	0.60375	33.0	0.62026
13.5	0.58660	23.5	0.60462	33.5	0.62103
14.0	0.58753	24.0	0.60548	34.0	0.62180
14.5	0.58846	24.5	0.60634	34.5	0.62257
15.0	0.58938	25.0	0.60719	35.0	0.62333
15.5	0.59030	25.5	0.60805	35.5	0.62408
16.0	0.59122	26.0	0.60889	36.0	0.62483
16.5	0.59214	26.5	0.60973	36.5	0.62557
17.0	0.59305	27.0	0.61057	37.0	0.62631
17.5	0.59396	27.5	0.61141	37.5	0.62704
18.0	0.59487	28.0	0.61223	38.0	0.62777
18.5	0.59577	28.5	0.61306	38.5	0.62849
19.0	0.59667	29.0	0.61388	39.0	0.62921
19.5	0.59757	29.5	0.61469	39.5	0.62992

**Table 3:** The mean absolute percentage error of the determination of the thermal conductivity of mPCM slurries.

Mass Fraction of Microcapsules	MAPE (%)			
	Water		Ergolid	
	PCM Solid	PCM Liquid	PCM Solid	PCM Liquid
4.3% mPCM	2.1	6.8	2.1	5.1
8.6% mPCM	3.7	8.6	3.4	4.8
12.9% mPCM	7.1	12.2	3.1	9.7
17.2% mPCM	10.0	14.7	1.4	11.9
21.5% mPCM	12.1	17.9	2.4	21.5
25.8% mPCM	13.9	22.0	4.6	23.2
30.1% mPCM	15.6	25.4	8.4	28.2
34.4% mPCM	18.4	28.0	11.1	28.1
38.7% mPCM	20.9	29.0	13.7	38.3
43.0% mPCM	24.7	28.0	-	-

## Data Availability

The data presented in this study are available on request from the corresponding author.
